# Supplementary material for: A Longitudinal Observational Study to Monitor the Outpatient–Caregiver Dyad in a Rehabilitation Hospital: Sociodemographic Characteristics and the Impact of Cognitive and Functional Impairment
Source: Brain Sci. 2025 Dec 10;15(12):1316. doi: 10.3390/brainsci15121316 (PMC12730887; doi:10.3390/brainsci15121316)
Supplement: Supplementary file 1 [file brainsci-15-01316-s001.zip › brainsci-3989715-supplementary.pdf]

Supplementary material

Table S1. Neuropsychological and functional assessment scores and interpretation

Test	*Construct*	*Scores Interpretation*
**MMSE**	Cognitive impairment	MMSE < 18.3 = cognitive impairment [1]
**ACE-R**	Cognitive impairment	ACE-R ≤ 1 = cognitive impairment [2]
**EQ-5D**	Quality of life questionnaire	High score = low quality of life
**EQ VAS**	Quality of life (VAS)	High score = high quality of life
**PHQ-4**	Distress	PHQ-4 ≥ 3 = distress symptoms
**FSQ-SF**	Caregiver's burden	FSQ-SF >6 = high caregivers' burden
**BADL**	Basic functional autonomy	High score = adequate autonomy
**IADL**	Instrumental functional ability	High score = adequate independence
**NPI-Q**	Neuropsychiatric symptoms	High score = more severe symptoms

[1] A patient's MMSE score <18.3 was used as the cut-off for inclusion in Dementia Group, which comprised individuals with a severe form of cognitive decline attributable to dementia.

[2] ACE-R scores of ≤1 combined with MMSE scores >22 were used as the cut-offs for inclusion in MCI Group, which comprised individuals with a mild form of cognitive decline attributable to MCI.
